# Order Space-Based Morphology for Color Image Processing

**DOI:** 10.3390/jimaging9070139

**Published:** 2023-07-07

**Authors:** Shanqian Sun, Yunjia Huang, Kohei Inoue, Kenji Hara

**Affiliations:** Department of Media Design, Faculty of Design, Kyushu University, 4-9-1, Shiobaru, Minami-ku, Fukuoka 815-8540, Japan; sunshanqian@outlook.com (S.S.); yuichi0507@163.com (Y.H.); hara@design.kyushu-u.ac.jp (K.H.)

**Keywords:** mathematical morphology, color image processing, order space, noise removal, exponentially weighted averaging, log-sum-exp, softmax, fuzzy

## Abstract

Mathematical morphology is a fundamental tool based on order statistics for image processing, such as noise reduction, image enhancement and feature extraction, and is well-established for binary and grayscale images, whose pixels can be sorted by their pixel values, i.e., each pixel has a single number. On the other hand, each pixel in a color image has three numbers corresponding to three color channels, e.g., red (R), green (G) and blue (B) channels in an RGB color image. Therefore, it is difficult to sort color pixels uniquely. In this paper, we propose a method for unifying the orders of pixels sorted in each color channel separately, where we consider that a pixel exists in a three-dimensional space called order space, and derive a single order by a monotonically nondecreasing function defined on the order space. We also fuzzify the proposed order space-based morphological operations, and demonstrate the effectiveness of the proposed method by comparing with a state-of-the-art method based on hypergraph theory. The proposed method treats three orders of pixels sorted in respective color channels equally. Therefore, the proposed method is consistent with the conventional morphological operations for binary and grayscale images.

## 1. Introduction

Mathematical morphology is a theory that originated in the 1960s for treating the shape of objects in images based on set theory and lattice theory. Mathematical morphology has a wide range of applications in digital image processing. At the present time, there are a large number of theoretical and practical studies on mathematical morphology, where the development of basic techniques for binary and grayscale images is almost complete. For a comprehensive introduction to morphological image analysis, one may refer to the book by Soille [1].

Unlike the case of binary or grayscale images, mathematical morphology for color images and higher-dimensional images is still under study because of the difficulty and uncertainty of ordering vector data, such as color vectors or pixels, in color images. That is, there is no canonical ordering of pixels in color images, and an arbitrary choice of order function that assigns an order to any pixel will provide good results for some problems or images and bad results for others.

Recently, a number of morphological techniques for color images have been presented to overcome the above difficulty. Sartor and Weeks  [2] proposed a method based on the (C−Y) color space, which has the luminance coordinate (*Y*) and the chromatic coordinates (R−Y,B−Y), where a generalized distance between a color and reference color is used for ordering color vectors. For computing the generalized distance, one requires a 3×3 covariance matrix. They also provided a brief introduction to lattice theory. Angulo also proposed a method to use a reference color for computing color distance, where the distance-based and lexicographical approaches are generalized in RGB, LSH and Lab color spaces [3]. d’Ornellas and Costa proposed an ordering relation for colors that is based on the natural ordering of spectra [4]. As a result, they derived a color-model-independent approach for color mathematical morphology. However, in compensation for the independency, their method requires reconstructing the fundamental spectrum from a conventional 3D color vector. Benavent et al. proposed a method based on the color histogram of a quantized color image [5], where the pixels are sorted on the basis of the frequency recorded in the histogram. In their method, the results may be affected by the number of quantized colors. Comer and Delp defined erosion and dilation for color morphology using marginal ordering, which uses only one component of a vector for ordering pixels, and reduced ordering, which uses a dimensionality reduction function for ordering three-dimensional pixel data [6]. Zhao also proposed a method for color morphology based on marginal ordering, where no new color artifacts are produced [7]. Van de Gronde and Roerdink pointed out that the false color problem lies in violating certain invariances, and demonstrated the effectiveness of hue-invariant and rotation-invariant methods [8]. Gimenez and Evans [9] analyzed the convex color sieve (CCS) by Gibson et al. [10] and the vector area morphology sieve (VAMS) by Evans [11] algorithmically and experimentally. Evans also described a chapter devoted to color area morphology scale spaces [12]. Burgeth et al. extended morphological maximum and minimum operations to tensor-valued settings by employing Loewner ordering for symmetric matrices [13]. Naegel and Passat extended the gray-level definition of component trees to multi-value images, and compared some strategies for color image processing based on component trees in the color image filtering and color document binarization fields [14]. Tushabe and Wilkinson proposed a color filtering method using max-tree representation, where a color image is converted into a grayscale image that is then subjected to the conventional filtering techniques, and demonstrated three restitution mechanisms that reconstruct the filtered image back into color: nearest color, nearest neighbor and mean of parent approaches [15]. Lezoray et al. proposed a method for the graph-based ordering of color vectors, and demonstrated the proposed mathematical morphology operations in RGB, IHSL [16], $L^{*}a^{*}b^{*}$ and CIECAM02 [17] color models [18]. Wang et al. proposed color morphological operators on a hypergraph to avoid the loss of details caused by fixed structuring elements [19]. The hypergraph-based method has two parameters for controlling spatial and color similarities between pixels.

In this paper, we propose a method for morphologically operating color images on the basis of an order space, which is a three-dimensional space with the axes of orders of pixels in three color channels. Specifically, we take the RGB (red, green and blue) color channels of RGB color images as a typical example, and sort the pixels in each channel separately. As a result, we obtain a triplet of orders for each pixel, which is then represented as a point in the order space. After that, we compute a single order from the obtained triplet of orders by a function from 3D to 1D, and then apply morphological operations based on the single order of pixels. In our experiments, we compere the proposed method with Wang’s hypergraph-based method [19], and demonstrate the performance of the proposed method in a noise-removal task. Moreover, we present the fuzzy version of the proposed order space-based method, which improves the performance further.

Since a color image is a stack of three grayscale images corresponding to three color channels, it would be consistent with the conventional morphological operation for binary and grayscale images to take into account the triplet of orders of pixels in the color version of the morphological operations. Therefore, the proposed order space-based method can be a plausible candidate for the extension of a grayscale morphology to a color one.

The rest of this paper is organized as follows: Section 2 briefly summarizes binary and grayscale morphological operations. Section 3 proposes the order space-based morphological operations for color images. Section 4 proposes an exponentially weighted averaging method for fuzzifying the order space-based method. Section 5 shows the experimental results obtained from a standard image dataset. Section 6 discusses the results obtained in Section 5. Finally, Section 7 concludes this paper.

## 2. Morphology for Binary and Grayscale Image Processing

In this section, we briefly summarize the basics of mathematical morphology for binary and grayscale images in preparation for the subsequent sections. In the following definitions of morphological operations, since binary images can be regarded as a special case of grayscale images, we focus on grayscale morphology [20].

**Figure 1:**
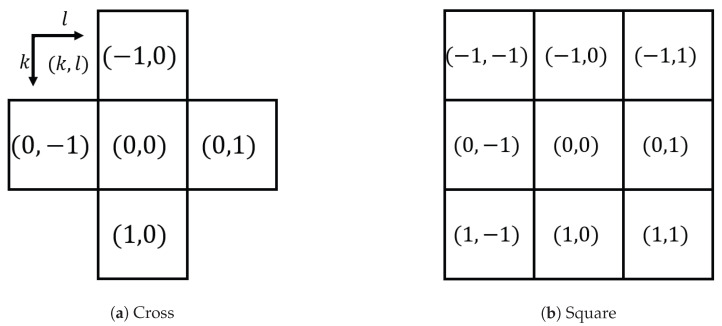
The 3×3 structuring elements: (**a**) The 3×3 ‘Cross’ structuring element is defined by a set of relative coordinates as S={(−1,0),(0,−1),(0,0),(0,1),(1,0)}. (**b**) The 3×3 ‘Square’ structuring element is defined by a set of relative coordinates as S={(−1,−1),(−1,0),(−1,1),(0,−1),(0,0),(0,1),(1,−1),(1,0),(1,1)}.

Let $G=[g_{ij}]$ be a grayscale image, where $g_{ij}\in \left\{0,1,\ldots ,L-1\right\}$ for i=1,2,…,m and j=1,2,…,n denotes the pixel value at the *i*th row and *j*th column in *G* with *m* rows and *n* columns, where L−1 denotes the maximum pixel value, e.g., $L=256=2^{8}$ for an 8-bit grayscale image. The grayscale pixel values $g_{ij}=0$ and $g_{ij}=L-1$ denote black and white pixels, respectively. To perform a morphological operation on *G*, we need a structuring element *S*, which is a set of relative coordinates, e.g., a 3×3 ‘Cross’ (or ‘Rhombus’) structuring element S={(−1,0),(0,−1),(0,0),(0,1),(1,0)} and a ‘Square’ one S={(−1,−1),(−1,0),(−1,1),(0,−1),(0,0),(0,1),(1,−1),(1,0),(1,1)}, which are illustrated in Figure 1. We assume that all structuring elements used in this paper are point-symmetric, which is a reasonable assumption for isotropic image processing tasks and simplifies the definitions of morphological operations as follows. (In a formal description, dilation is defined as the Minkowski set addition by a given structuring element. On the other hand, erosion is defined as the Minkowski set subtraction by the reflection of the structuring element. With the symmetric structuring elements, we can skip the reflection step.)

The dilation of *G* by a symmetric structuring element *S* is defined as

(1) $$D\left(G,S\right)=\left[d_{ij}\right],\;d_{ij}=\max_{(k,l)\in S}\left\{g_{i+k,\;j+l}\right\},$$

and the erosion of *G* by the same *S* is defined as

(2) $$E\left(G,S\right)=\left[e_{ij}\right],\;e_{ij}=\min_{(k,l)\in S}\left\{g_{i+k,\;j+l}\right\},$$

where we note that if i+k∉{1,2,…,m} or j+l∉{1,2,…,n}, then the corresponding value $g_{i+k,j+l}$ is excluded from the candidates for output pixel.

The above dilation and erosion operations change the mean brightness of a given image. To compensate for such bias caused by dilation and erosion operations, they are often combined with each other as follows: the opening of *G* by *S* is defined as

(3) O(G,S)=D(E(G,S),S),

and the closing of *G* by *S* is defined as

(4) C(G,S)=E(D(G,S),S),

i.e., the opening operation is composed of an erosion followed by a dilation, and the closing operation is composed of a dilation followed by an erosion. The opening and closing operations are also combined with each other as follows: the open–closing of *G* by *S* is defined as

(5) OC(G,S)=C(O(G,S),S),

and the close–opening of *G* by *S* is defined as

(6) CO(G,S)=O(C(G,S),S),

i.e., the open–closing operation is composed of an opening followed by a closing, and the close–opening operation is composed of a closing followed by an opening (the definitions of the open–closing and close–opening operations are convertible into each other as mentioned by Schevenels and Sigmund [21]).

Here, we note that if L=2, then these definitions are reduced to those for binary images.

## 3. Order Space-Based Morphology for Color Image Processing

The above morphological operations for binary and grayscale images include the maximum and minimum selection, which is based on the assumption that the pixels in an image can be sorted in ascending or descending order of their pixel values. For a binary or grayscale image, it is easy to sort the pixels by their values. However, for a color image, it is not easy because each color pixel has three values, e.g., red, green and blue components, the orders of which may conflict with each other. Therefore, sorting each color component separately frequently causes undesirable colors.

In this section, we propose a method for morphologically processing color images in an appropriate manner, which can alleviate the drawback of the naive separable method.

Let $F=[f_{ij}]$ be an RGB color image, where $f_{ij}=\left[f_{ij}^{R},f_{ij}^{G},f_{ij}^{B}\right]$ for i=1,2,…,m and j=1,2,…,n denotes the RGB color vector at the position (i,j) in *F*, where $f_{ij}^{R},f_{ij}^{G}$ and $f_{ij}^{B}$ denote the red (R), green (G) and blue (B) components of the pixel $f_{ij}$, respectively. Then, we can sort the color components covered by a structuring element in ascending order of their values, e.g., $f_{i+k\left(a_{1}^{R}\right),\;j+l\left(a_{1}^{R}\right)}^{R}\leq f_{i+k\left(a_{2}^{R}\right),\;j+l\left(a_{2}^{R}\right)}^{R}\leq \cdots \leq f_{i+k\left(a_{5}^{R}\right),\;j+l\left(a_{5}^{R}\right)}^{R}$ for the R component with a structuring element *S* with five pixels, as shown in Figure 1a, where $a_{1}^{R},a_{2}^{R},\ldots ,a_{5}^{R}$ denote the sorted indices of pixels covered by *S*, and $k(a_{1}^{R})$ and $l(a_{1}^{R})$ denote the vertical and horizontal relative coordinates of the pixel corresponding to $a_{1}^{R}$, respectively.

**Figure 2:**
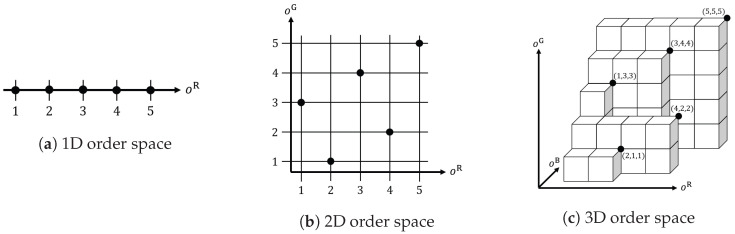
Illustrations of 1D, 2D and 3D order spaces for RGB color images: (**a**) Five pixels are arranged in a line (or a one-dimensional order space) according to the ascending order of their R components. For binary and grayscale images, we can consider the similar 1D order space. (**b**) If we take into account R and G components simultaneously, then we have a two-dimensional order space onto which five pixels are located according to their orders. (**c**) If we take into account R, G and B components simultaneously, then we have a three-dimensional order space onto which five pixels are located according to their orders.

Let $\xi =a_{\eta }^{R}$ (for η=1,2,…,5) be a function where a pixel index ξ is related to an order η given by the above sorting procedure. Then, we consider the inverse function of $\xi =a_{\eta }^{R}$ as $\eta =o_{\xi }^{R}={\left(a^{R}\right)}_{\xi }^{-1}$, where an order η is related to a pixel index ξ. That is, each pixel ξ has the corresponding order η. According to the order, we can arrange the five pixels on an axis as shown in Figure 2a, where each black point denotes a pixel ξ, which is placed at the position designated by the order η.

Similarly, we can sort the set of pixels in the ascending order of the G components to have the sorted indices $a_{1}^{G},a_{2}^{G},\ldots ,a_{5}^{G}$ satisfying $f_{i+k\left(a_{1}^{G}\right),\;j+l\left(a_{1}^{G}\right)}^{G}\leq f_{i+k\left(a_{2}^{G}\right),\;j+l\left(a_{2}^{G}\right)}^{G}\leq \cdots \leq f_{i+k\left(a_{5}^{G}\right),\;j+l\left(a_{5}^{G}\right)}^{G}$. We also consider the inverse function of $\xi =a_{\eta }^{G}$ as $\eta =o_{\xi }^{G}={\left(a^{G}\right)}_{\xi }^{-1}$. Then, we can plot the five pixels on a two-dimensional coordinate system with the axes $o^{R}$ and $o^{G}$ as shown in Figure 2b, where we note that no two pixels are aligned with one another vertically and horizontally.

By sorting the B components, we also have the sorted indices $a_{1}^{B},a_{2}^{B},\ldots ,a_{5}^{B}$ satisfying $f_{i+k\left(a_{1}^{B}\right),\;j+l\left(a_{1}^{B}\right)}^{B}\leq f_{i+k\left(a_{2}^{B}\right),\;j+l\left(a_{2}^{B}\right)}^{B}\leq \cdots \leq f_{i+k\left(a_{5}^{B}\right),\;j+l\left(a_{5}^{B}\right)}^{B}$, which are summarized as a function $\xi =a_{\eta }^{B}$ for η=1,2,…,5, the inverse function of which is given by $\eta =o_{\xi }^{B}={\left(a^{B}\right)}_{\xi }^{-1}$. Then, we can plot each pixel on a three-dimensional coordinate system with the axes $o^{R},\;o^{G}$ and $o^{B}$ as shown in Figure 2c. We call the space determined by the above coordinate system *order space*. As a result, an RGB color pixel identified by an index ξ is mapped to a point on the order space as $\left(o_{\xi }^{R},o_{\xi }^{G},o_{\xi }^{B}\right)$. This is a natural consequence of the generalization of the basics of morphological operations for binary and grayscale images, since there are no advantages and disadvantages among the three color channels. Therefore, it is reasonable to construct morphological operations for color images on the basis of the 3D order space.

Algorithm 1 summarizes the procedure for mapping from an RGB color space to the order space. In Algorithm 1, line 3, the function ‘argsort’ sorts the arguments $f_{1}^{X},f_{2}^{X},\ldots ,f_{\left|S\right|}^{X}$ in ascending order as $f_{a_{1}^{X}}^{X}\leq f_{a_{2}^{X}}^{X}\leq \cdots \leq f_{a_{\left|S\right|}^{X}}^{X}$, and returns their indices $a_{1}^{X},a_{2}^{X},\ldots ,a_{\left|S\right|}^{X}$, where |S| denotes the number of elements of *S*. Each value of the coordinates in the order space is given at line 5.
**Algorithm 1** Mapping from RGB color space to order space**Require:** 
a set of pixels $\left\{f_{i+k,\;j+l}=\left[f_{i+k,\;j+l}^{R},\;f_{i+k,\;j+l}^{G},\;f_{i+k,\;j+l}^{B}\right]\;|\;\left(k,l\right)\in S\right\}$**Ensure:** 
a set of coordinates in order space $\{\left(o_{\xi }^{R},o_{\xi }^{G},o_{\xi }^{B}\right)\;|\;\xi \in \{1,2,\ldots ,|S|\}\}$ 1:Assign serial numbers ξ∈{1,2,…,|S|} to all pixels in the required set to have a set of re-indexed pixels $\{f_{\xi }=\left[f_{\xi }^{R},\;f_{\xi }^{G},\;f_{\xi }^{B}\right]\;|\;\xi \in \{1,2,\ldots ,|S|\}\}$. 2:**for** X∈{R,G,B} **do** 3:    $\left[a_{1}^{X},a_{2}^{X},\ldots ,a_{\left|S\right|}^{X}\right]=\mathrm{argsort}\left(f_{1}^{X},f_{2}^{X},\ldots ,f_{\left|S\right|}^{X}\right)$ 4:    **for** ξ∈{1,2,…,|S|} **do** 5:        $o_{a_{\xi }^{X}}^{X}=\xi$ 6:    **end for** 7:**end for** 8:Return $\{\left(o_{\xi }^{R},o_{\xi }^{G},o_{\xi }^{B}\right)\;|\;\xi \in \{1,2,\ldots ,|S|\}\}$

In the 3D order space, an RGB color pixel with an index ξ is represented by a triplet $\left(o_{\xi }^{R},o_{\xi }^{G},o_{\xi }^{B}\right)$. To define dilation and erosion for an RGB color image, we need to reduce the triplet $\left(o_{\xi }^{R},o_{\xi }^{G},o_{\xi }^{B}\right)$ to a singlet $o_{\xi }$. If we have $o_{\xi }^{R}\leq o_{\xi^{\prime }}^{R},\;o_{\xi }^{G}\leq o_{\xi^{\prime }}^{G}$ and $o_{\xi }^{B}\leq o_{\xi^{\prime }}^{B}$ for two pixels ξ and $\xi^{\prime }$, then we can assign an order satisfying $o_{\xi }\leq o_{\xi^{\prime }}$. For example, the following equations for obtaining a singlet $o_{\xi }$ from the triplet $\left(o_{\xi }^{R},o_{\xi }^{G},o_{\xi }^{B}\right)$ satisfy this condition:

(7) $$o_{\xi }^{S}=o_{\xi }^{R}+o_{\xi }^{G}+o_{\xi }^{B},$$

(8) $$o_{\xi }^{P}=o_{\xi }^{R}o_{\xi }^{G}o_{\xi }^{B},$$

(9) $$o_{\xi }^{M}=\mathrm{med}\left\{o_{\xi }^{R},o_{\xi }^{G},o_{\xi }^{B}\right\},$$

where ‘med’ means to select the median value from a given set of elements. We chose these equations here for their simplicity and easy implementation. Other choices may include weighted average, mode and nonlinear transformations of the order triplet. For example, let us consider three pixels in 3D order space: a=(1,2,3),b=(3,1,2) and c=(2,3,1). Then, the above three Equations (7)–(9) give the same orders $o^{S}=1+2+3=6,\;o^{P}=1\times 2\times 3=6$ and $o^{M}=\mathrm{med}\left\{1,2,3\right\}=2$ for a,b and *c*. To assign different orders to different points in 3D order space, we need more sophisticated equations than the above.

**Figure 3:**
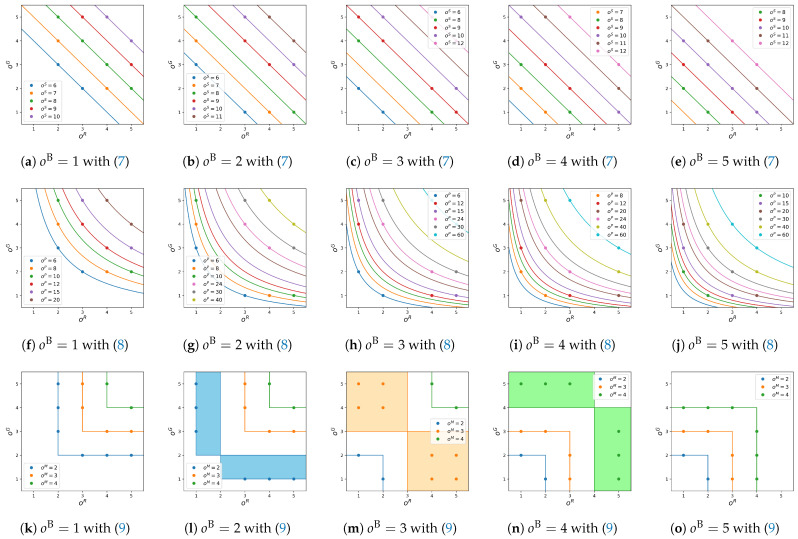
Contour lines in three order spaces corresponding to (7)–(9): (**a**–**e**) $o_{\xi }^{S}$ given by (7) with fixed $o^{B}=1,2,\ldots ,5$, respectively. (**f**–**j**) $o_{\xi }^{P}$ given by (8) with fixed $o^{B}=1,2,\ldots ,5$, respectively. (**k**–**o**) $o_{\xi }^{M}$ given by (9) with fixed $o^{B}=1,2,\ldots ,5$, respectively. In (**l**–**n**), the contour lines for $o^{M}=2,\;3$ and 4 are spread over the plane $o^{B}=2,\;3$ and 4 as illustrated by light blue, light orange and light green areas, respectively.

Figure 3 shows the contour lines of $o_{\xi }^{S},\;o_{\xi }^{P}$ and $o_{\xi }^{M}$ in their order spaces, where the top, middle and bottom rows correspond to $o_{\xi }^{S},\;o_{\xi }^{P}$ and $o_{\xi }^{M}$, respectively. In each row, the five figures from left to right show cross sections for $o^{B}=1,2,\ldots ,5$. In each figure, the horizontal and vertical axes denote $o^{R}$ and $o^{G}$. For example, in Figure 3a, feasible orders are denoted by color points, and contour lines for fixed values of $o^{S}=6,7,\ldots ,10$ are denoted by color lines with the corresponding colors. For fixed $o^{S}$ and $o^{B}$, we have a straight line $o^{G}=\left(o^{S}-o^{B}\right)-o^{R}$ with slope −1. On the other hand, in the middle row of Figure 3, for fixed $o^{P}$ and $o^{B}$, we have a hyperbola $o^{G}=\left(o^{P}/o^{B}\right)/o^{R}$. In the bottom row of Figure 3, we have no analytical expressions for the contour lines. However, we can draw them as shown in this figure. It is interesting that there are contour ‘areas’ as shown in Figure 3l–n with light blue, light orange and light green colors.

Once a function from $\left(o_{\xi }^{R},o_{\xi }^{G},o_{\xi }^{B}\right)$ to $o_{\xi }$ is given like in (7)–(9), we can define the dilation and erosion of *F* by *S* as follows:

(10) $$D\left(F,S\right)=\left[d_{ij}\right],\;d_{ij}=f_{\xi^{\max}},\;\xi^{\max}=\arg\max_{\xi \in \{1,2,\ldots ,|S|\}}o_{\xi },$$

(11) $$\begin{array}{c} E\left(F,S\right)=\left[e_{ij}\right],\;e_{ij}=f_{\xi^{\min}},\;\xi^{\min}=\arg\min_{\xi \in \{1,2,\ldots ,|S|\}}o_{\xi }. \end{array}$$

Next, we combine the above dilation and erosion operations for defining opening and closing operations as follows:

(12) O(F,S)=D(E(F,S),S),

(13) C(F,S)=E(D(F,S),S).

Here, we define an image $Z=[z_{\xi }]$ whose pixel value $z_{\xi }$ denotes the order $o_{\xi }$ of the corresponding pixel of *F*. Then, the above morphological operations correspond to the grayscale morphological operations applied to *Z*. Therefore, idempotence, increasingness and (anti-)extensiveness hold for the application of morphological opening and closing operations to *Z* as well as the conventional opening and closing operations.

Combining the above opening and closing operations, we can define open–closing and close–opening operations as follows:

(14) OC(F,S)=C(O(F,S),S),

(15) CO(F,S)=O(C(F,S),S),

which are consistent with the definitions of the corresponding operations for binary and grayscale images summarized in the previous section.

**Figure 4:**
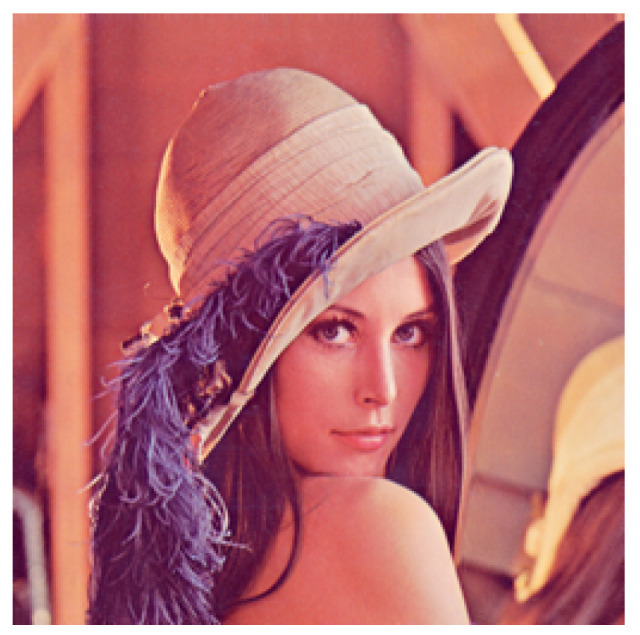
Original color image selected from SIDBA [22].

**Figure 5:**
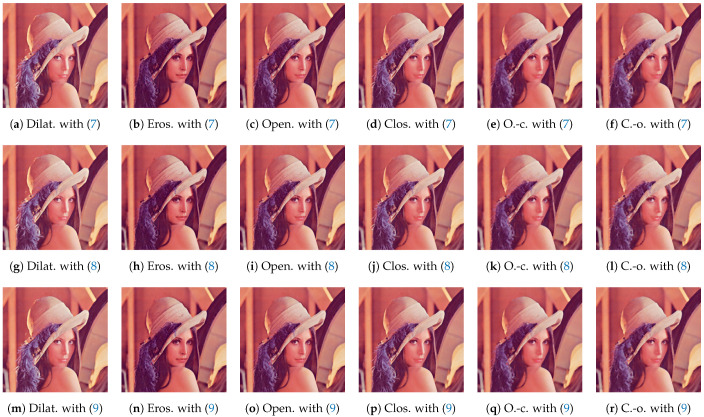
Order space-based morphological operations for a color image in Figure 4: The **top**, **middle** and **bottom** rows show the results with (7)–(9), respectively. The six columns from left to right show the results of dilation (Dilat.), erosion (Eros.), opening (Open.), closing (Clos.), open–closing (O.-c.) and close–opening (C.-o.) operations, respectively.

We applied the proposed order space-based morphological operations to a color image in Figure 4, and obtained the images in Figure 5, where the top, middle and bottom rows were obtained with $o^{S},\;o^{P}$ and $o^{M}$ in (7)–(9), and the six columns from left to right show the results of dilation, erosion, opening, closing, open–closing and close–opening operations, respectively. We observe that the three kinds of ordering, $o^{S},\;o^{P}$ and $o^{M}$, give similar output images to each other.

## 4. Fuzzy Morphological Operations by Exponentially Weighted Averaging

In this section, we extend the above order space-based morphological operations, which are crisp operations, to fuzzy ones by introducing an exponentially weighted averaging method.

Assume that an order $o_{\xi }$ for ξ∈{1,2,…,|S|} is obtained from a set of triplets $\left\{\left(o_{\xi }^{R},o_{\xi }^{G},o_{\xi }^{B}\right)\right\}$. Then, we define a fuzzy dilation by introducing an exponentially weighted averaging to (10) as follows:

(16) $$D^{\mathrm{FUZ}}\left(F,S,\alpha \right)=\left[d_{ij}^{\mathrm{FUZ}}\right],\;d_{ij}^{\mathrm{FUZ}}=\frac{\sum_{\xi =1}^{\left|S\right|}\exp\left(\alpha o_{\xi }\right)f_{\xi }}{\sum_{\xi =1}^{\left|S\right|}\exp\left(\alpha o_{\xi }\right)},$$

where exp denotes the exponential function defined by $\exp\left(x\right)=e^{x}$, where *e* is a constant called Euler’s number and α is a positive constant for controlling the fuzziness. Here, we note that the second equation in (16) includes the softmax function [23], which is the gradient of the log-sum-exp function [24]. Therefore, the equation can be rewritten as

(17) $$d_{ij}^{\mathrm{FUZ}}=\arg\min_{d}\;\sum_{\xi =1}^{\left|S\right|}\frac{\partial }{\partial o_{\xi }}\left(\log\sum_{\nu =1}^{\left|S\right|}\exp\left(\alpha o_{\nu }\right)\right){∥f_{\xi }-d∥}^{2},$$

which explicitly describes the relationship between the exponentially weighted averaging and the log-sum-exp function, where $\partial /\partial o_{\xi }$ denotes a partial derivative with respect to $o_{\xi }$ and ∥·∥ denotes the Euclidean norm. This expression accepts vectors $f_{\xi }$ and *d* of any dimension. Therefore, we can fuzzify morphological operations for not only color images but also binary and grayscale images by the proposed method.

Similarly, we can define a fuzzy erosion as follows:

(18) $$E^{\mathrm{FUZ}}\left(F,S,\alpha \right)=\left[e_{ij}^{\mathrm{FUZ}}\right],\;e_{ij}^{\mathrm{FUZ}}=\frac{\sum_{\xi =1}^{\left|S\right|}\exp\left(-\alpha o_{\xi }\right)f_{\xi }}{\sum_{\xi =1}^{\left|S\right|}\exp\left(-\alpha o_{\xi }\right)}.$$

Combining these operations, we have fuzzy opening and closing operations as follows:

(19) $$O^{\mathrm{FUZ}}\left(F,S,\alpha \right)=D^{\mathrm{FUZ}}\left(E^{\mathrm{FUZ}}\left(F,S,\alpha \right),S,\alpha \right),$$

(20) $$C^{\mathrm{FUZ}}\left(F,S,\alpha \right)=E^{\mathrm{FUZ}}\left(D^{\mathrm{FUZ}}\left(F,S,\alpha \right),S,\alpha \right).$$

From these, we further define fuzzy open–closing and close–opening operations as follows:

(21) $$OC^{\mathrm{FUZ}}\left(F,S,\alpha \right)=C^{\mathrm{FUZ}}\left(O^{\mathrm{FUZ}}\left(F,S,\alpha \right),S,\alpha \right),$$

(22) $$CO^{\mathrm{FUZ}}\left(F,S,\alpha \right)=O^{\mathrm{FUZ}}\left(C^{\mathrm{FUZ}}\left(F,S,\alpha \right),S,\alpha \right).$$

The value of α is empirically determined in the following section.

## 5. Experimental Results

**Figure 6:**
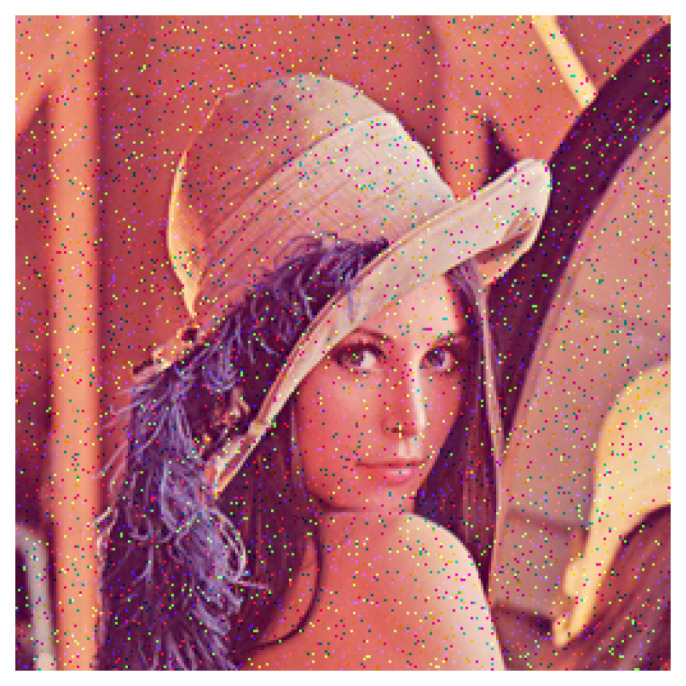
Noisy input image including 10% impulse noise, where 6553 out of 256 × 256 = 65,536 pixels are corrupted with impulse noise.

In this section, we show the experimental results of noise removal from color images by the proposed order space-based morphological operations, which are compared with the state-of-the-art method of Wang et al. [19]. Figure 6 shows a noisy input image including 10% impulse noise.

**Figure 7:**
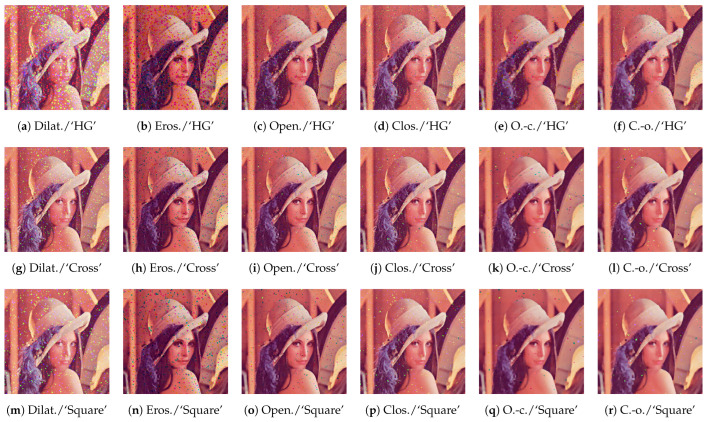
Noise removal results using Wang’s method (**top** row) and the proposed order space-based morphological operations (**middle** and **bottom** rows) with $o^{S}$ in (7) for a noisy image in Figure 6, where their subcaptions denote the ‘operation/structuring element’ used for computing respective images: The top row shows the results of Wang’s method, where ‘HG’ indicates that the structuring elements are given by hypergraphs. The middle and bottom rows show the results of the proposed method with ‘Cross’ and ‘Square’ structuring elements shown in Figure 1. The six columns from left to right show the results of dilation (Dilat.), erosion (Eros.), opening (Open.), closing (Clos.), open–closing (O.-c.) and close–opening (C.-o.) operations, respectively.

Figure 7 shows the output images by Wang’s hypergraph-based method [19] and the proposed method with ‘Cross’ and ‘Square’ structuring elements in Figure 1a and Figure 1b, respectively, where the order $o^{S}$ in (7) is used. Wang’s method has two parameters, μ and β, which are set as μ=250 and β=1 by adjusting for this noise removal task through preliminary experiments. The top row in this figure shows the results of Wang’s method with a structural hypergraph denoted by ‘HG’ as an alternative to structuring elements for the dilation, erosion, opening, closing, open–closing and close–opening operations, the output images of which are arranged from left to right in columns, where we observe that the dilation Figure 7a and erosion Figure 7b tend to enlarge impulse noise, but the following operations Figure 7c–f can suppress the noise effectively. The middle and bottom rows in Figure 7 show the results of the proposed method with ‘Cross’ and ‘Square’ structuring elements shown in Figure 1a and Figure 1b, respectively. The 3×3 ‘Square’ structuring element corresponds to the structuring hypergraph with β=1 in Wang’s method in terms of size.

Table 1 shows the values of the peak signal-to-noise ratio (PSNR) [25] between the output images in Figure 7 and the original noise-free image in Figure 4, where Wang’s method with the ‘HG’ structuring element achieves the highest PSNR 21.59 dB by the close–opening operation. On the other hand, the proposed method with ‘Cross’ and ‘Square’ structuring elements achieves 25.14 and 25.83 dB by open–closing and close–opening, respectively.

**Figure 8:**
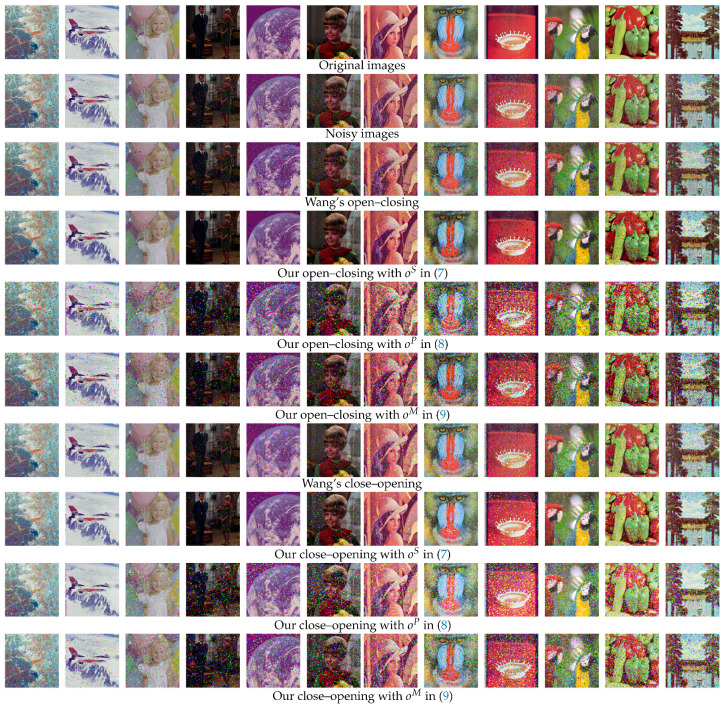
Noise removal results for 12 color images in the SIDBA dataset [22]: The original images in the top row receive impulse noise with densities varying from 10% (the leftmost two columns) to 60% (the rightmost two columns), as shown in the second row. The third and subsequent rows show the corresponding output images from the open–closing and close–opening operations in Wang’s method and the proposed method with $o^{S},\;o^{P}$ and $o^{M}$ in (7)–(9), respectively.

Figure 8 shows the noise removal results on the SIDBA image dataset [22], where the top row lists all 12 original color images, which are corrupted by impulse noise as shown in the second row, where the noise density increases from the left to right columns. We adopted open–closing and close–opening operations for removing the noise on the basis of the observation in Table 1, where those operations achieved higher PSNR values than the other morphological operations. In this figure, we compared the proposed method based on the ordering $o^{S},\;o^{P}$ and $o^{M}$ in (7)–(9), computed with the 3×3 ‘Square’ structuring element in Figure 1b with Wang’s method using the parameters μ=250 and β=1.

**Figure 9:**
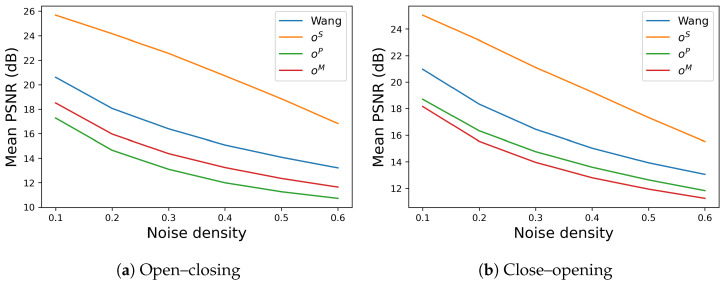
Mean PSNR vs. noise density on SIDBA dataset [22]: (**a**) Open–closing operations of Wang’s and the proposed methods are compared with each other, where the proposed method with $o^{S}$ (orange line) outperforms Wang’s method (blue line). (**b**) Similarly, close–opening operations are compared, where the proposed method with $o^{S}$ (orange line) also outperforms Wang’s method (blue line).

The performance of noise removal is evaluated by the mean PSNR among the 12 different images as summarized in Figure 9, where Figure 9a,b show the results of open–closing and close–opening, respectively. In each graph, the vertical and horizontal axes denote the mean PSNR among the 12 images and the density of impulse noise varying from 10% to 60%, respectively, and the line colors, blue, orange, green and red correspond to Wang’s method and the proposed methods with $o^{S},\;o^{P}$ and $o^{M}$, respectively. In both graphs, Wang’s method (blue line) obtains higher values of mean PSNR than the proposed methods with $o^{P}$ and $o^{M}$ (green and red lines), and the proposed method with $o^{S}$ (orange line) outperforms Wang’s method.

**Figure 10:**
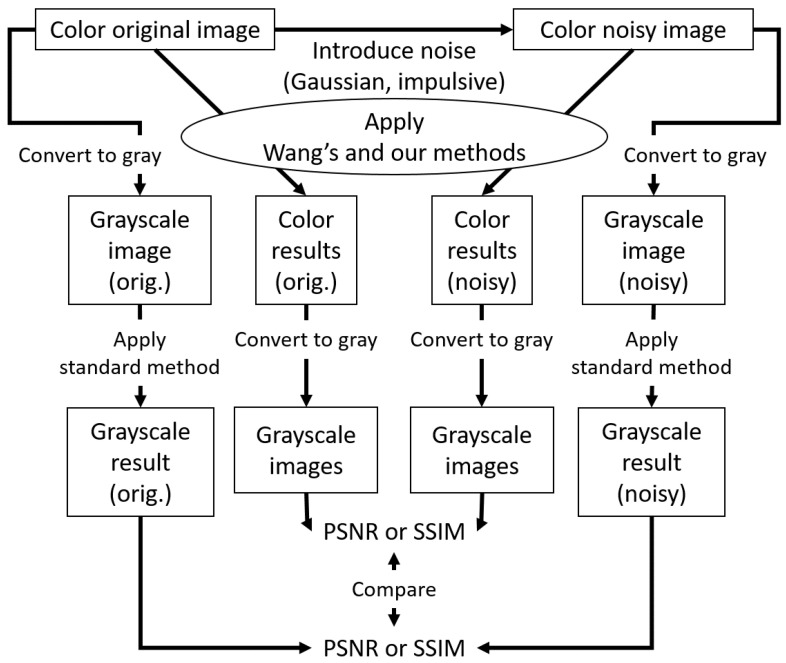
Comparison of grayscale and color morphological operations with Gaussian and impulse noises. In this flow chart, grayscale conversions are followed by grayscale morphological operations (outer routes), and color morphological operations are followed by grayscale conversions (inner routes). Finally, the corresponding grayscale images are compared by computing PSNR or SSIM.

**Figure 11:**
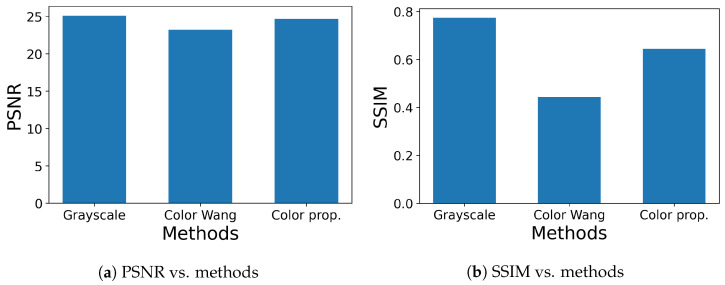
Results of Gaussian noise removal, where Gaussian noise with mean 0 and standard deviation 30 are added to the original images in Figure 8: (**a**) PSNR values are compared for grayscale, Wang’s and our open–closing operations. (**b**) SSIM values are compared for grayscale, Wang’s and our open–closing operations.

We also compared the performance of grayscale and color morphological operations as illustrated in Figure 10, where a color original image (top left box) is corrupted by Gaussian or impulse noise to produce a noisy image (top right box). Both images are converted to grayscale images through the outside clockwise and counterclockwise routes. Then, the standard method for grayscale images is applied to those grayscale images (bottom leftmost and rightmost boxes). On the other hand, color original and noisy images pass through inner routes, where Wang’s and our methods for color images are applied to them. Then, the resultant images are converted to grayscale images (bottom middle two boxes). Finally, we compute PSNR or the structural similarity index measure (SSIM) [26] for the resultant grayscale images processed by the same method. Figure 11 shows the results of Gaussian noise removal with the SIDBA dataset [22], where the vertical axes denote Figure 11a PSNR and Figure 11b SSIM, and the horizontal axes denote the compared methods. In this figure, the grayscale operation achieved the highest PSNR and SSIM values, and the proposed method outperformed Wang’s method.

**Figure 12:**
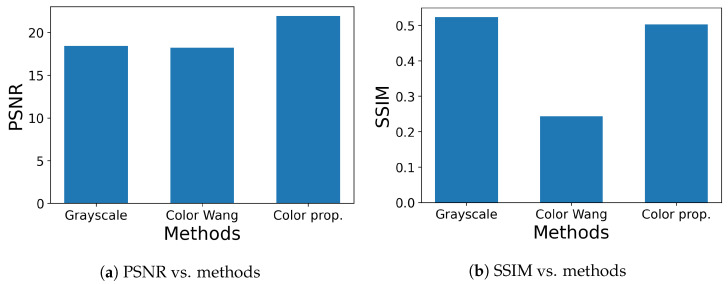
Results of impulse noise removal, where 50% impulse noise is added to the original images in Figure 8: (**a**) PSNR values are compared for grayscale, Wang’s and our open–closing operations. (**b**) SSIM values are compared for grayscale, Wang’s and our open–closing operations.

On the other hand, Figure 12 shows the results of impulse noise removal, where the settings of axes are the same as Figure 11. In Figure 12a, the proposed method achieved the highest PSNR value, while in Figure 12b, the grayscale operation achieved the highest SSIM value. The reason why the grayscale operation achieves higher values than color ones is speculated to be that the conversion from color to gray has a noise-reduction effect. This speculation is investigated in Table 2, where color noisy images are compared with grayscale noisy images by PSNR and SSIM for Gaussian and impulse noises. The values of the image quality measure for R, G and B components are improved by grayscale conversion for all combinations of noises and measures. This result shows that the grayscale conversion has a noise-reduction effect.

In terms of the color morphological operations in this experiment, the proposed method achieved better PSNR and SSIM values than Wang’s method.

**Figure 13:**
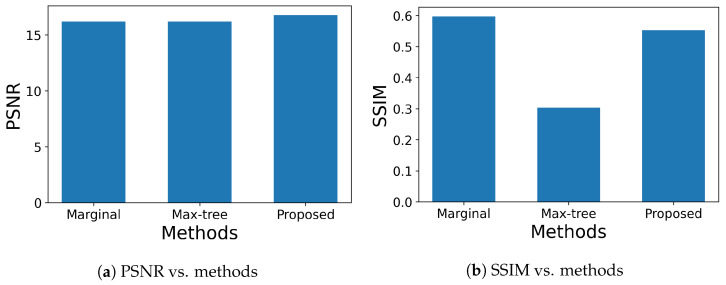
Comparison of marginal open–closing, Max-tree-based area open–closing and the proposed order space-based open–closing for impulse noise removal: (**a**) PSNR values are compared for marginal, Max-tree-based area and the proposed open–closing operations. (**b**) SSIM values are compared for marginal, Max-tree-based area and the proposed open–closing operations.

For impulse noise removal, it is well known that simple marginal processing based on marginal ordering works well, although it can generate false colors. Thus, we compared the proposed method with the marginal open–closing and Max-tree-based area open–closing proposed by Tushabe and Wilkinson [15], where we adopted an area threshold of T=150 and a nearest color (NC) approach for image restoration as recommended in their paper. Figure 13 shows the results of noise removal, where 12 color images in the SIDBA dataset [22] were corrupted by 50% impulse noise, and we applied the three open–closing operations to those noisy images. The performance of noise removal was evaluated with PSNR and SSIM, which are shown in Figure 13a and Figure 13b, respectively. In Figure 13a, the proposed method achieved the highest PSNR value among the three methods. On the other hand, in Figure 13b, the simple marginal method achieved the highest SSIM value, which matches the above knowledge that the marginal method removes impulse noise well.

**Figure 14:**
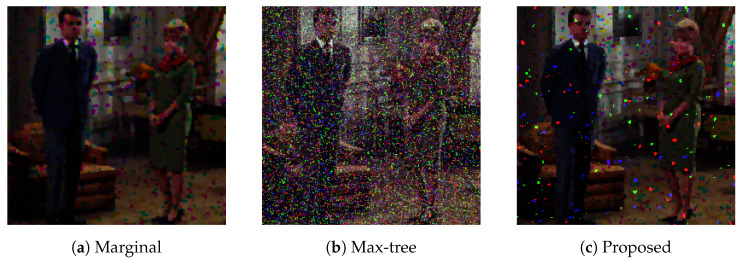
Output images obtained by (**a**) marginal open–closing, (**b**) Max-tree-based area open–closing and (**c**) the proposed order space-based open–closing.

Figure 14 shows the output images computed by the three methods, where the image in Figure 14a given by the simple marginal open–closing operation appears to be the best among three output images. However, the image in Figure 14a includes new colors that do not exist in the input image. This violates a desirable property that color morphological operations should have, i.e., no new colors should be introduced [27].

**Figure 15:**
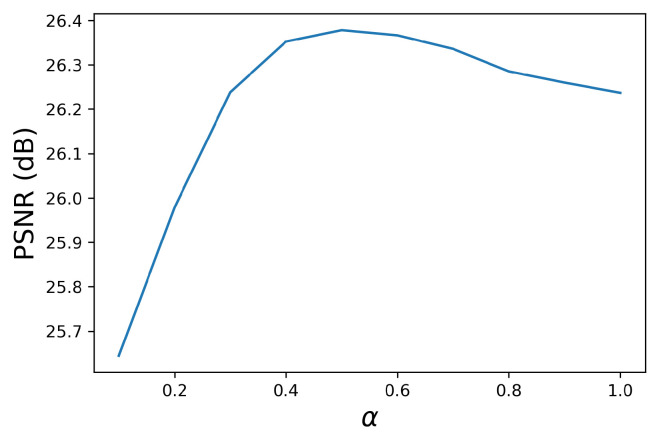
PSNR vs. α for controlling the fuzziness of the proposed fuzzy morphological operations. This figure shows that α=0.5 gives the highest PSNR value in the proposed fuzzy open–closing operation applied to the noisy image in Figure 6.

Next, we show the experimental results for the fuzzy morphological operations described in Section 4. Here, we would like to determine the value of α in the fuzzy operations with the open–closing operation, which outperforms the close–opening operation as shown in Figure 9; that is, the orange line in Figure 9a exceeds that in Figure 9b. Figure 15 shows the PSNR values for different values of α varying from 0.1 to 1.0, where we applied the fuzzy open–closing in (21) to the noisy image in Figure 6. We observe that α=0.5 achieves the highest PSNR value, and use α=0.5 below.

**Figure 16:**
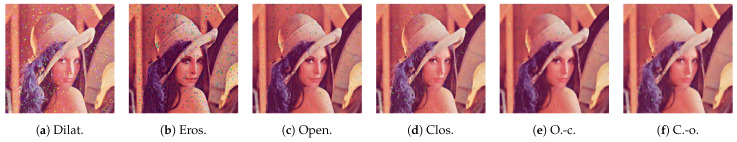
Noise removal results by fuzzy morphological operations with α=0.5 applied to the noisy image in Figure 6. The order of the output images (**a**–**f**) by six morphological operations, dilation (Dilat.), erosion (Eros.), opening (Open.), closing (Clos.), open–closing (O.-c.) and close–opening (C.-o.), is the same as that of Figure 7.

**Figure 17:**
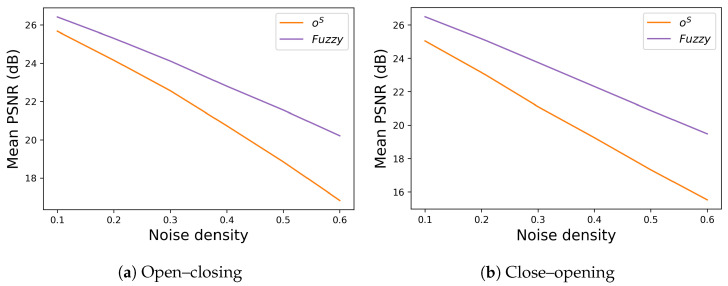
Comparison of mean PSNR between crisp and fuzzy morphological operations: (**a**) Crisp (orange line) and fuzzy (purple line) open–closing operations are compared, where the former is the same as the orange line in Figure 9a. (**b**) Similarly, crisp (orange line) and fuzzy (purple line) close–opening operations are compared, where the former is the same as the orange line in Figure 9b.

Figure 16 shows the results of the proposed fuzzy morphological operations defined in (16)–(22). A visual comparison between Figure 16 and Figure 7 demonstrates the effectiveness of the proposed fuzzification, and Figure 17 shows the effectiveness quantitatively by comparing the mean PSNR values computed with the SIDBA dataset [22] between crisp and fuzzy operations, where the vertical and horizontal axes denote the mean PSNR and noise density as well as the settings from Figure 9, and Figure 17a,b which show the results of open–closing and close–opening operations, respectively. For both operations, the fuzzy ones denoted by purple lines outperform the crisp ones denoted by orange lines.

## 6. Discussion

In the above experimental results, we demonstrated the effectiveness of the proposed order space-based morphological operations, which are compared with Wang’s method based on hypergraph theory [19]. Generally, color images have three color channels, e.g., red, green and blue channels for RGB color images, and we can sort the pixel values in each channel separately. Therefore, it would be appropriate to consider three orders derived from the three channels in morphological operations for color images. The proposed method is a method by which the obtained three orders, which form a 3D order space, can be unified into a single order of pixels in a suitable manner for a given application. In this paper, we presented three ways of unifying three orders corresponding to three color channels into a single order, i.e., $o^{S},\;o^{P}$ and $o^{M}$ defined in (7), (8) and (9), respectively. In the application of morphological operations to noise removal demonstrated in Section 5, Wang’s method [19] achieved better performance than the proposed method with $o^{P}$ and $o^{M}$, but the remaining option $o^{S}$ outperformed Wang’s method, which shows the potential of the proposed order space-based method. The ways of unifying three orders are not restricted to the three methods presented in this paper. Investigating better ways to unify plural orders into one will be a promising future research direction.

We also extended the proposed order space-based morphological operations to their fuzzy version, where we proposed a method for fuzzifying morphological operations by exponentially weighted averaging. Conventional morphological operations for binary and grayscale images include maximum and minimum selection. The max function for selecting the maximum value from a set of scalar values can be approximated by the log-sum-exp function [24], which has a relationship with fuzzy techniques [28]. However, it is difficult to use the log-sum-exp function directly for fuzzifying morphological operations for color images because of the dimensional discrepancy between scalar and vector data. On the other hand, the proposed exponentially weighted averaging method can be used for both scalar and vector data. Therefore, we can also fuzzify conventional morphological operations for binary and grayscale images by the proposed method. In the above experiments, we demonstrated the effectiveness of the fuzzification of the order space-based morphological operations in the noise-removal application. The applicability of the proposed fuzzification method to any dimensional data will expand its application areas into the area of multidimensional data processing.

## 7. Conclusions

In this paper, we proposed a method for applying morphological operations to color images. The proposed method is based on a three-dimensional (3D) order space, which has three coordinate axes corresponding to the orders of pixels in three color channels, e.g., R (red), G (green) and B (blue) color channels for RGB color images. That is, a color pixel is represented by a point on the 3D space specified by the set of orders of pixels sorted in R, G and B color channels, separately. Then, the triplet was shrunk to a single order by an appropriate function, e.g., sum, product and median functions were investigated in this paper. We conducted experiments on noise removal with a standard image dataset, and demonstrated that the sum function achieved the best performance among the three functions and a state-of-the-art method based on hypergraph theory by Wang et al. [19]. We can consider other options for the shrinkage functions and the structuring elements in morphological operations that may further improve the performance.

We also proposed an exponentially weighted averaging method for fuzzifying the order space-based morphological operations. As a result, the noise removal performance was further improved by the fuzzification for both open–closing and close–opening operations, both of which achieved better performance than dilation, erosion, opening and closing operations in a conventional crisp situation.

Our future work will include the extension of the proposed method for color image processing to multidimensional data processing, which will also be fuzzified by the proposed exponentially weighted averaging method to improve the performance.

## Figures and Tables

**Table 1:** PSNR for output images in Figure 7 with ‘HG’, ‘Cross’ and ‘Square’ structuring elements (SEs). In each row, the highest value is boldfaced.

SE	Dilation	Erosion	Opening	Closing	Open-Closing	Close-Opening
‘HG’ [19]	15.31	16.22	20.11	20.52	20.88	**21.59**
‘Cross’	18.82	18.80	22.96	23.11	**25.14**	25.13
‘Square’	17.79	17.73	23.67	23.97	25.74	**25.83**

**Table 2:** Comparison of the quality of noisy images used in Figure 10 between color and gray. In each row, the highest value is boldfaced.

Noise	Quality Measure	R	G	B	Gray
Gaussian	PSNR	18.90	19.06	19.03	**22.71**
Gaussian	SSIM	0.306	0.350	0.315	**0.417**
Impulse	PSNR	12.16	11.94	12.01	**16.63**
Impulse	SSIM	0.118	0.144	0.122	**0.183**

## Data Availability

Not applicable.

## Abbreviations

The following abbreviations are used in this manuscript:

RGB	Red, green and blue
PSNR	Peak signal-to-noise ratio
SSIM	Structural similarity index measure
SIDBA	Standard Image DataBAse
SE	Structuring element
